# Highly sensitive proximity mediated immunoassay reveals HER2 status conversion in the circulating tumor cells of metastatic breast cancer patients

**DOI:** 10.1186/1477-5956-9-75

**Published:** 2011-12-15

**Authors:** Phillip Kim, Xinjun Liu, Tani Lee, Limin Liu, Robert Barham, Richard Kirkland, Glen Leesman, Anne Kuller, Belen Ybarrondo, Shi-Chung Ng, Sharat Singh

**Affiliations:** 1 Department of Research & Development, Oncology, Prometheus Laboratories, 9410 Carroll Park Dr., San Diego, CA 92121, USA

**Keywords:** Companion diagnostics, Collaborative enzyme enhanced reactive-immunoassay, Metastatic breast cancer, Circulating tumor cells, HER2 conversion

## Abstract

**Background:**

The clinical benefits associated with targeted oncology agents are generally limited to subsets of patients. Even with favorable biomarker profiles, many patients do not respond or acquire resistance. Existing technologies are ineffective for treatment monitoring as they provide only static and limited information and require substantial amounts of tissue. Therefore, there is an urgent need to develop methods that can profile potential therapeutic targets with limited clinical specimens during the course of treatment.

**Methods:**

We have developed a novel proteomics-based assay, Collaborative Enzyme Enhanced Reactive-immunoassay (CEER) that can be used for analyzing clinical samples. CEER utilizes the formation of unique immuno-complex between capture-antibodies and two additional detector-Abs on a microarray surface. One of the detector-Abs is conjugated to glucose oxidase (GO), and the other is conjugated to Horse Radish Peroxidase (HRP). Target detection requires the presence of both detector-Abs because the enzyme channeling event between GO and HRP will not occur unless both Abs are in close proximity.

**Results:**

CEER was able to detect single-cell level expression and phosphorylation of human epidermal growth factor receptor 2 (HER2) and human epidermal growth factor receptor 1 (HER1) in breast cancer (BCa) systems. The shift in phosphorylation profiles of receptor tyrosine kinases (RTKs) and other signal transduction proteins upon differential ligand stimulation further demonstrated extreme assay specificity in a multiplexed array format. HER2 analysis by CEER in 227 BCa tissues showed superior accuracy when compared to the outcome from immunohistochemistry (IHC) (83% vs. 96%). A significant incidence of HER2 status alteration with recurrent disease was observed via circulating tumor cell (CTC) analysis, suggesting an evolving and dynamic disease progression. HER2-positive CTCs were found in 41% (7/17) while CTCs with significant HER2-activation without apparent over-expression were found in 18% (3/17) of relapsed BCa patients with HER2-negative primary tumors. The apparent 'HER2 status conversion' observed in recurrent BCa may have significant implications on understanding breast cancer metastasis and associated therapeutic development.

**Conclusion:**

CEER can be multiplexed to analyze pathway proteins in a comprehensive manner with extreme specificity and sensitivity. This format is ideal for analyzing clinical samples with limited availability.

## Background

Breast cancer is a collection of diseases with distinct histopathological features and diverse prognostic outcomes. As the field rapidly progresses towards understanding the diverse biology of breast cancers, we are presented with a range of treatment options to treat this malignancy. Owing to the differences in response to treatment, the search for a tool to differentiate breast cancer subtypes and to predict response when patients are newly diagnosed or when the disease has recurred has been intense.

A classic example is the HER2-positive breast cancers that comprise approximately 25-30% of breast cancers [1,2]. HER2 is a receptor member of the ErbB receptor tyrosine kinase (RTK) family that is activated by phosphorylation after dimerization with other receptor member partners to initiate pathway signaling. Over-expression of HER2 triggers cell proliferation and disease progression, and HER2-positive BCa have a higher recurrence rate and reduced survival [1]. With the advent of HER2-targeted therapies, most notably trastuzumab, the natural progression of HER2-positive breast cancers can be dramatically blunted [3,4]. Therefore, HER2 overexpression is accepted as a strong predictive marker for clinical benefits from trastuzumab [5]. However, only approximately 50% of HER2-positive patients initially respond to trastuzumab-complemented treatments while the rest show inherent resistance and can metastasize to distant sites. Even the patients who demonstrate a dramatic initial response to trastuzumab eventually develop resistance [6]. If there were a way to prospectively predict the course of breast cancer progression and strategically segregate the responders from the non-responders, it would eliminate uncertainty in treatment and save valuable time providing most effective evidence-based therapeutic outcome.

Multi-target assessments of gene expression in normal and abnormal tissues have expanded our understanding of the pathophysiology of many diseases including breast cancers. While mRNA profiling can provide valuable biological information, its clinical potential may be limited because the mRNA levels may or may not correspond to the expressed protein levels. Despite these limitations, advances made in basic and translational research have resulted in the incorporation of genomic technologies into clinical use for complex diseases such as cancer, thus paving the way for new genomic-based patient management [7,8]. Multiplexed genomic-analysis matured due to the exquisite sensitivity and specificity of molecular technologies based on sequence-specific target amplification processes.

In contrast, proteomic-based methods have not yet developed into a practical multiplexed format. Most current protein-based applications are based on traditional IHC principles, which are semi-quantitative at best and require a substantial amount of sample. The more successful clinical application of proteomics technologies awaits better sensitivity and specificity. Additionally, an efficient proteomics-based diagnostic platform must be able to differentiate the level of total protein expression from the degree of protein activation as the activated state of the proteins reflects their impact on cellular functions.

One of the most widely used current clinical applications of proteomic assessments for therapeutic/prognostic outcome is with the detection of HER2 protein expression in BCa patients using IHC. However, this method has technical limitations with analytical sensitivity, target specificity, capacity to multiplex, and subjectivity in image interpretation [9,10]. Furthermore, significant discordance between the results of HER2 studies performed in different laboratories has been reported [11]. Hence fluorescence in situ hybridization (FISH) technology is currently used to detect HER2 gene amplification when the IHC-based results are ambiguous. A staged use of both technologies is used to determine patient eligibility for trastuzumab [12]. Although HER2-IHC and HER2-FISH are valuable for preliminary patient selection, neither test can accurately differentiate trastuzumab responders from non-responders. A further limitation of both these assay methods is their inability to determine the activation status of the HER2 protein. Therefore, there is a definite need for a proteomics-based method to identify unequivocally which HER2-positive breast cancer patients will respond to HER2-targeting agents. Such methods should be able to determine the functional state of HER2 along with the profile of its potential dimerization partners, in order to provide vital information for rational selection of the most effective therapy option. Another valuable characteristic of a versatile diagnostic test would be if it could molecularly evaluate breast cancer progression with high sensitivity and specificity on limited amounts of clinical material. As tumors are extremely heterogeneous, the tumor cells at the primary site of occurrence may not necessarily reflect the profile of the tumor cells in recurrent disease. A relevant source of tumor cells for capturing metastases of recurrent disease may be the CTCs found in peripheral blood [13-16]. Although sample volume may be limited, these provide valuable opportunities to perform a non-invasive "real-time liquid biopsy" on metastatic cancer patients.

**Figure 1:**
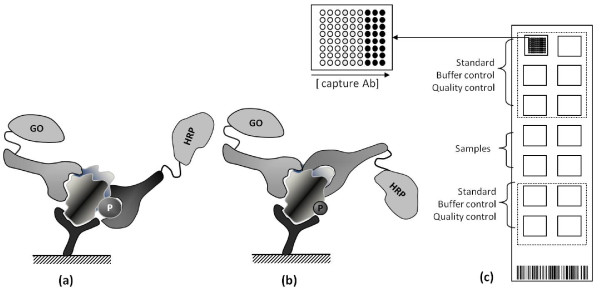
**Configuration of Collaborative Enzyme Enhanced Reactive-immunoassay (CEER)**. When target proteins are bound to specific capture antibodies printed on nitrocellulose surface after incubating with cell lysate, unbound non-target proteins are removed from the slide. One of the detector antibodies against an alternate epitope on captured target-protein is conjugated with GO. Binding of another detector antibody specific to phosphorylated sites (P) on target protein **(a) **or another non-overlapping epitope **(b) **conjugated with HRP completes the formation of immuno-complex necessary for signal generation and subsequent tyramide mediated signal amplification through GO-HRP enzyme channeling in the presence of glucose. The capture and detection antibodies were selected to minimize competition between them (*i.e*., all antibodies can simultaneously bind their corresponding epitope on the signal transduction protein). **(c) **A slide configuration for CEER is shown. Capture antibodies for each specific target protein are printed in triplicates in serial dilution. Each slide contains cell line controls for standard curve generation for accurate quantitation of samples on each slide run. Internal quality control samples are run on each slide to ensure the quality of data generated from each array-slide.

We have developed a novel microarray-based proteomic platform, Collaborative Enzyme Enhanced Reactive-immunoassay (CEER; Figure 1) that has ultra-high sensitivity and specificity due to its unique configuration. It can simultaneously detect the activation state of multiple signal transduction proteins at the single cell level with an analytical sensitivity of about 100 zeptomoles (or between 1 × 10^4 ^to 1 × 10^5 ^target molecules). Here we report the successful application of CEER to quantitate the total expression and the activation state of a number of RTKs including HER2 and other downstream signaling pathway proteins in several breast cancer cell lines, xenografts, and breast cancer clinical samples. We further present evidence that CEER can be successfully used to analyze CTCs from metastatic breast cancer patients that can aid treatment decisions for HER2-targeting agents. We demonstrate that novel biological aspects of breast cancer progression can be uncovered by directly analyzing clinical samples using the CEER technology.

## Results

### CEER can detect ErbB-RTK activation status at the single cell level in breast cancer cell lines

**Figure 2:**
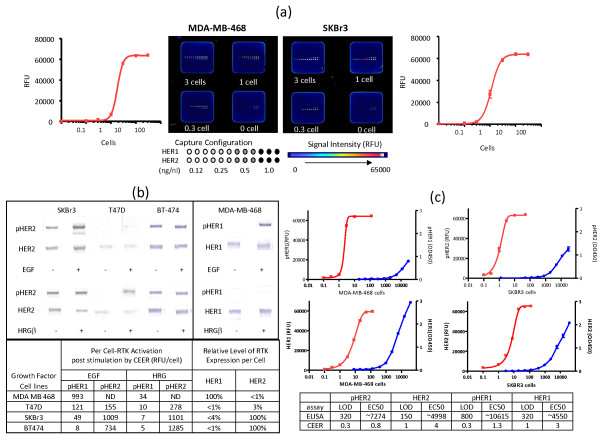
**CEER's single cell sensitivity and capacity for functional profiling of pathway proteins**. **(a) **The activation of HER1 and HER2 at a sensitivity level of a single cell in MDA-MB468 and SKBr3 respectively are shown above. These cell lines express approximately 1 to 2 × 10^6 ^HER1 or HER2 RTKs on their cell membrane per cell. Microarray slide images for 3 cells, 1 cell, 0.3 cell and negative control are shown above the cell titration curve. MDA-MB468 cells were treated with EGF to activate HER1 while HER2 is constitutively phosphorylated in SKBr3 cells. The cell amount on each pad was generated by serial dilution. Capture antibodies were printed with 500 pl per spot in triplicates in serial dilutions of 1.0 mg/ml, 0.5 mg/ml, 0.25 mg/ml and 0.125 mg/ml. Relative signal intensity scale is shown for reference. **(b) **The western blot data were generated from 12 μg of total protein per lane. The level of dominant RTK expression in each cell line was determined pre- and post- EGF or HRG stimulation. The CEER determined per-cell RTK activation (RFU/cell) level for each cell line is summarized. Non-detectable signals in each cell lines were indicated as ND in the table. **(c) **Detection of pHER1, pHER2, total HER1 and total HER2 in SKBR3 and MDA-MB-468 cells using CEER or ELISA assays are shown. Sensitivity of CEER and ELISA assays were shown by detection limit, which was defined as cell numbers when signal noise ratio (s/n) > 3. pHER1 in MDA-MB-468 cells (top left), pHER2 in SKBR3 cells (top right), HER1 in MDA-MB-468 cells (bottom left) and HER2 in SKBR3 cells (bottom right). Closed square (red): HER2s or HER1s (RFU) detected by CEER assay; Closed circle (blue): HER2s or HER1s (OD450) detected by ELISA assay.

CEER was used to detect the expression and activation (phosphorylation) of HER1 and HER2, receptor members of the ErbB-RTK family, at a sensitivity level of a single cell in breast cancer cell lines, MDA-MB-468 and SKBr3, respectively (Figure 2a). These cell lines have been well characterized for their ErbB-RTK expression status [17-21]. Although RTK expression is approximately 1 to 2 × 10^6 ^HER1 or HER2 receptors per cell in MDA-MB-468 and SKBr3, respectively, only subsets of these receptors are phosphorylated at any given instance. However, the small percentage of phosphorylated receptors in these cell lines is sufficient to drive downstream pathway activation and breast cancer cell proliferation [18]. Therefore, to efficiently detect HER1 and HER2 receptor activation at a single cell level in breast cancer cells, it is necessary to detect these subsets of phosphorylated receptors.

While HER2 over-expressing SKBr3 cells demonstrate constitutive HER2 activation, MDA-MB-468 cells need to be stimulated with HER1-specific EGF ligand to induce HER1 phosphorylation [17,18]. As expected and as shown in Figure 2b, differential activation of HER1 and HER2 occurs in cell lines expressing varying levels of ErbB receptor family members and when they are stimulated by either EGF (for direct HER1 stimulation *via *homo or heterodimerization) or HRG (for indirect stimulation of HER2 *via *heterodimerization with HER3). While MDA-MB-468 cells did not show any HER1 activation at basal (unstimulated) states, they showed significant HER1 phosphorylation upon EGF treatment (Figure 2b). Typically only 2-10% of highly expressed RTKs are phosphorylated upon ligand stimulation which equates to approximately 2 × 10^4 ^to 1 × 10^5 ^phosphorylated receptors per MDA-MB-468 or SKBr3 cell [18]. As shown in a cell titration experiment in Figure 2a, CEER enabled us to detect approximately 10^4 ^phosphorylation events in both MDA-MB-468 and SKBr3 cells, thus demonstrating that this assay is capable of producing single cell sensitivity. The RTK activation per cell upon growth factor treatment using CEER is also summarized in Figure 2b.

To demonstrate the sensitivity of CEER compared with a commonly used enzyme immunoassay, CEER and ELISA were used in a side-by-side comparison to detect phosphorylated HER1 (pHER1) and HER1 expression (total HER1) in MDA-MB-468 and pHER2 and total HER2 in SKBr3 serially diluted cell lysates. A signal sensitivity comparison is illustrated by plotting cell numbers of the two assays for pHER2, pHER1, HER2 and HER1 in test samples (Figure 2c). The detection limit was defined as the point at which the signal-to-noise ratio of cell numbers was > 3. The plots show that the cell titration curves of ELISA were shifted right by more than 2 logs and that the detection limit of ELISA ranged from 150-800 cells: pHER2 = 320 cells, HER2 = 150 cells, pHER1 = 800 cells, and HER1 = 320 cells. In contrast, the detection limit of the same analytes in the same samples was determined to be 1 cell or less using CEER. The EC50 value comparison for each analyte showed greater than 3 log sensitivity enhancement by CEER (Summary table in Figure 2c).

In order to quantitate the HER1 and HER2 expression levels in the test samples to get an idea of the level of sensitivity of CEER at the absolute receptor level, we used purified recombinant HER1 and HER2 proteins (R & D systems) as standards in an ELISA assay (data not shown). The detection range for HER1 was 313-10000 pg and 156-10000 pg for HER2 in an ELISA. Based on our assay results, we determined that there were approximately 0.6 pg HER1 molecules per MDA-MB-468 cell and 0.8 pg HER2 molecules per SKBr3 cell, respectively. Thus, one MDA-MB-468 cell contains ~ 2.0 × 10^6 ^HER1 receptors and one SKBr3 cell contains ~ 2.5 × 10^6 ^HER2 receptors, which is consistent with other published results [18,20,21]. This analysis also agrees with our previous calculations and reiterates that CEER is able to detect 10^4^-10^5 ^phosphorylated ErbB receptors per cell in the breast cancer systems.

### CEER can efficiently detect ErbB pathway activation in breast cancer systems that lack *HER2 *gene amplification

**Figure 3:**
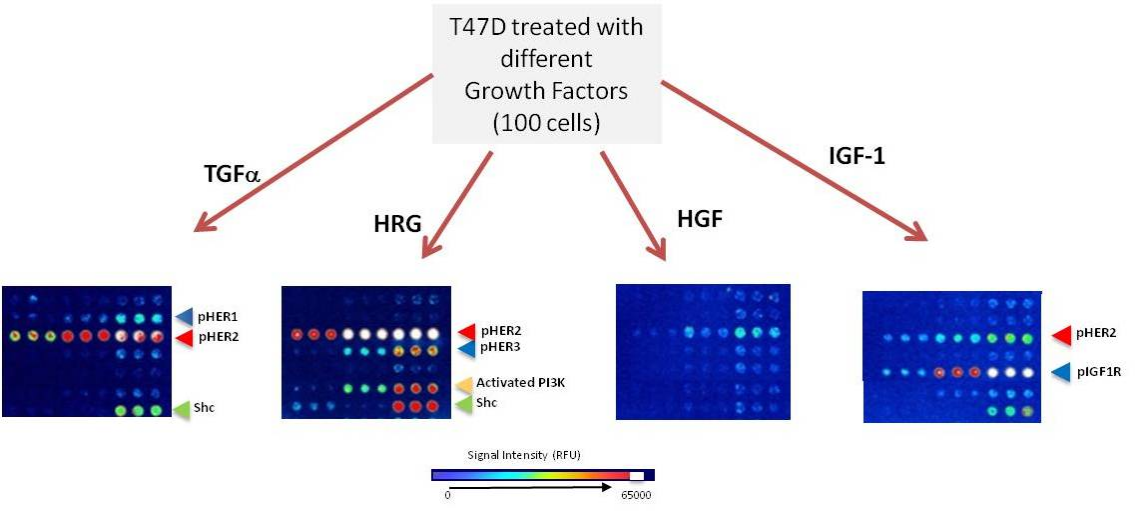
**Detection of ligand specific activation of different signal transduction pathway proteins**. T47D cells upon different ligand (TGFα, HRGβ or IGF1) treatment were analyzed for various RTK and subsequent downstream pathway protein activation.

Our studies thus far demonstrated the high sensitivity and specificity of the CEER format. While pHER2 was undetectable in MDA-MB-468 cells, they displayed 993 RFU/cell level of phosphorylated HER1 (pHER1) when stimulated with EGF (Figure 2b). In contrast, EGF treated SKBr3 cells displayed substantially lower level of pHER1 than pHER2 in CEER in agreement with their known relative ErbB receptor expression levels. We next wanted to interrogate the utility of CEER (both sensitivity and specificity) in breast cancer systems that lack overexpressed HER2. This would be important as many breast cancers do express HER2 but do not harbor overexpressed or gene-amplified HER2 status (IHC; 3+ or FISH; HER2/CEN17 > 2.2). We carried out the ErbB pathway analysis using CEER in T47D cells. T47D cells carry normal levels of HER2 (IHC based score of 0/1+) that are substantially lower than SKBr3 cells [22]; however, a significant level of HER2 phosphorylation was detected in this system when approximately 10 cells were analyzed in a CEER format. Differential ErbB receptor and pathway activation patterns were observed when these cells were stimulated with either EGF/TGFα or HRG ligands (Figure 2b and Figure 3). As T47D cells express a significantly higher level of HER3 than HER1, higher HER2 activation was observed when cells were activated with HRG *via *HER2-HER3 heterodimer formation. HRG treatment did not induce HER1 activation in this cell population demonstrating assay specificity. On the other hand, TGFα treatment of T47D cells resulted in both HER1 and HER2 activation through HER1-HER2 heterodimerization, although at a lower level than HRG mediated activation. In addition to HER1 and HER2, the differential phosphorylation patterns of other RTKs that interact with ErbB-RTKs and downstream pathway proteins were interrogated (Figure 3). Stronger activation of Shc was observed upon HRG treatment, and PI3K was only activated with HRG treatment, which is consistent with a HER3-mediated cellular signal transduction process. Upon insulin-like growth factor 1 (IGF1) treatment, T47D cells showed ligand-specific IGF1-receptor (IGF1R) phosphorylation along with some level of HER2 activation (Figure 3). Recent evidence from trastuzumab-resistant breast cancer cell lines suggests that functional heterodimerization of HER2 and IGF1R may contribute to trastuzumab resistance [23]. Binding of IGF1 to its receptor results in its auto-phosphorylation and subsequent activation of downstream proteins, especially the PI3K pathway, ultimately resulting in increased cell proliferation. Overall, these studies demonstrated the ErbB ligand-dependent specificity of CEER in several breast cancer cell lines.

We next evaluated if CEER could be utilized in *in vivo *samples to profile HER1 and HER2 in tumor tissues obtained through (Fine Needle Aspirates) FNAs from breast cancer xenografts (Table 1) with varying degrees of ErbB-RTK expression [17-19,24]. BT474 is known to have *HER2 *gene amplification and overexpresses the HER2 protein. On the other hand, MDA-MB-435 and MDA-MB-231 do not express gene amplified *HER2 *but MDA-MB-231 expresses HER1 at high levels while both HER1 and HER2 are expressed at much lower levels in MDA-MB-435. We detected 4619 RFU/μg and 3813 RFU/μg of pHER1 and pHER2, respectively, in MD-MB-231 xenograft tissue. A substantially higher level of pHER2 (59890 RFU/μg) and moderate levels of pHER1 (6557 RFU/μg, due to co-expression with amplified HER2) was detected in FNA samples obtained from a BT474 xenograft. Very low HER1 and HER2 activation (724 RFU/μg and 1301 RFU/μg, respectively) were detected in FNAs obtained from MDA-MB-435 xenograft (Table 1). Although MDA-MB-435 has been used as a model for metastatic human breast cancer, interestingly it has recently been revealed that this cell line resembles melanoma according to gene expression profiling; hence, our results were consistent with what has been reported previously [25]. Taken together, our findings from the xenograft-FNA model systems are concordant with the parent cell line HER2 profiles. This demonstrates that the CEER technology can be used to detect activation of ErbB receptors in samples obtained from both *HER2 *gene amplified and non-amplified breast cancer systems including limited amounts of tumor tissue isolated through minimally invasive FNA procedures.

**Table 1:** CEER can be utilized to profile tumor cells present in FNAs

	*HER2 *Gene Amplification	HER1 (RTK/cell)	HER2 (RTK/cell)	pHER1 (RFU/μg)	pHER2 (RFU/μg)
BT474	Yes	3 × 10^4^	2.2 × 10^6^	6557	59860

MDA-MB-435	No	1.3 × 10^4^	2.2 × 10^3^	724	1301

MDA-MB-231	No	1.3 × 10^5^	6 ×10^4^	4619	3813

The levels of HER1 and HER2 expression in cell lines used for xenograft models are shown as number of RTK expressed per cell (BT474, MDA-MB-435 and MDA-MB-231 [12-15,22]). The degree of RTK activation in FNA samples obtained from xenograft models are shown in RFU per μg of tissue lysate.

### Comparison of CEER with IHC in primary human breast tumors

Currently, IHC and FISH are widely used tests for HER2 detection in clinical breast cancer tissues. Although there are some controversies concerning the measurement of HER2 amplification in breast cancers, it is conventionally accepted that trastuzumab is only effective in a subset of breast cancers, those with elevated HER2 expression as assessed by these diagnostic assays. There are specific guidelines recommended by the American Society of Clinical Oncology (ASCO) and College of American Pathologists (CAP) that define HER2 positive and HER2-negative breast cancers based on IHC and FISH assays [26]. To test the concordance of CEER and IHC results, CEER-HER2 expression analysis was performed on primary BCa tissues obtained from 227 patients with various IHC-HER2 statuses. All tissues were procured with a minimum of 50% tumor content.

Although reporting expression/activation levels of RTKs in RFUs is useful, results from CEER analysis need to be normalized against standard controls before they can be reliably utilized in clinical environments. Hence, we developed algorithms for converting RFU values into Computed Units (CU), a standard functional unit based on cell line controls with known HER1 and HER2 expression. For each slide, a standard curve of serially diluted cell lysate was prepared from MDA-MB-468 (HER1-positive) and SKBr3 (HER2-positive) cell lines as shown in Figure 1c. This made it possible to obtain normalized levels of HER1 and/or HER2 expression and the degree of phosphorylation in each sample against standard cell lines. Hence, a sample with 1 CU of HER1 expression has a RFU value equivalent to RFU value of 1 standard reference MDA-MB-468 cell. As reference cells have 1~2 × 10^6 ^HER1 or HER2 receptors per cell with approximately 10% phosphorylated receptors, 1 CU represents the expression of 1~2 × 10^6 ^RTKs or 1~2 × 10^5 ^phosphorylated RTKs. The limit of detection (LOD) value by CU was determined to be less than 1 CU for both pHER1 and pHER2 in a CEER assay.

**Figure 4:**
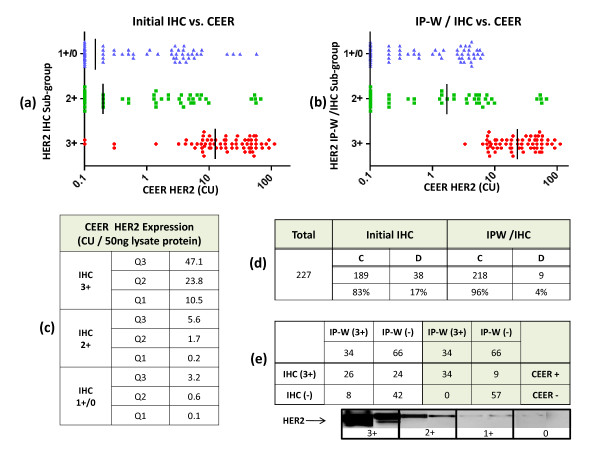
**Concordance of CEER with IHC in primary breast tumors**. The distribution of HER2 expression based on CEER in each IHC sub-group are shown in **(a)**. The quantitative distribution within each sub-group is shown in **(c)**. There were 17% (38/227) discordant calls between IHC and CEER HER2 status (C - concordance, D - discordance). The outcome of IP-W analysis of 100 samples (including all with discordant calls) are summarized in **(e)**. The concordance between CEER and IP-W adjusted HER status are shown in **(b) **and **(d)**. Representative IP-W images for each sub-group are shown in **(e)**.

The breast cancer tissue samples were segregated based on their HER2 status determined using IHC procedures based on ASCO/CAP guidelines. The HER2 expression levels (in 50 ng of total lysate protein) in each IHC sub-group were subsequently determined by CEER and are shown in Figure 4a. Most samples within the HER2-IHC 3+ sub-group had significant level of HER2 detected by CEER while a substantially lower level of HER2 expression was detected in the other sub-groups (2+, 1+ and 0); their quartile distribution is summarized in Figure 4c. Based on this CEER HER2 breakdown, we observed 17% discordance between the HER2 status as determined by CEER and IHC as shown in Figure 4d, and this finding is similar to previously reported findings [26-28]. A total of 100 samples (a subset of 227) from the various HER2-IHC sub-groups including all samples with a discordant outcome between CEER and IHC were further analyzed for their HER2 expression by immunoprecipitation western blot (IP-W). An immunoprecipitation assay combined with a western blot with HER2-specific antibodies is more accurate and quantitative as compared to an IHC assay. In this subset analysis, when compared to IP-W, IHC HER2 status revealed 68% concordance to IP-W with 23.5% (8/34) false negative calls (Figure 4e, left panels). When HER2 status between CEER and IP-W were compared, we found a 91% concordance between them with no false negative call by CEER (Figure 4e, right panels). As patients with a false negative call would not receive HER2-targeted therapy, it is absolutely critical for a technology to have minimal false negative calls to be effective in providing clinical utility. When CEER-HER2 values were compared to IP-W corrected IHC HER2 status (IPW/IHC), the concordance rate between CEER and HER2-IHC improved from 83% to 96% (Figure 4d). All discordant calls between IP-W and CEER were due to the higher sensitivity of CEER; corrected IHC vs. CEER HER2 status are shown in Figure 4b. These data demonstrate the superiority of CEER in terms of its high sensitivity and specificity in breast cancer clinical samples with a significantly reduced false negative rate frequently observed with HER2-IHC.

### Expression of activated HER2 in circulating tumor cells reveals an evolving and dynamic signaling pathway in metastatic breast cancers

CTCs are gaining significant attention as they provide an opportunity to perform non-invasive and temporally-relevant tumor assessments [13,14,16,29,30]. CTC analysis from metastatic cancer patients provide an additional opportunity to study how representative they are of the metastatic cancers in terms of their specific pathway activation as CTC presence has been associated with worse progression-free and overall survival. However, in order to expand the utility of CTCs as prognostic indicators of metastatic disease and treatment response, the technology for interrogating their pathway activation profiles must be highly sensitive, specific, reproducible, standardized and related to clinical outcomes.

**Figure 5:**
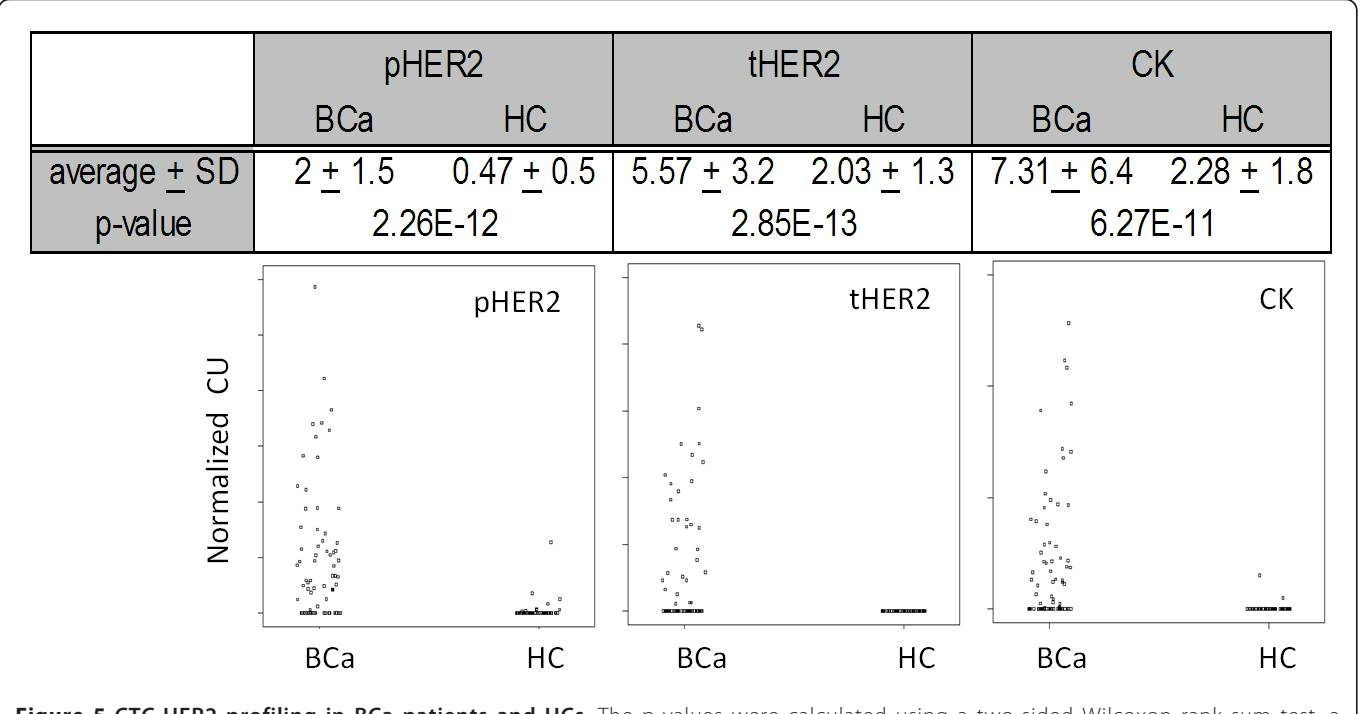
**CTC-HER2 profiling in BCa patients and HCs**. The p-values were calculated using a two-sided Wilcoxon rank sum test, a nonparametric test similar to a t-test. All of the comparisons show a statistically significant difference (p < 0.01). To determine pHER2 and tHER2 status, the background and reference values was subtracted (normalized CU). Scatter plots of normalized CU for pHER2, tHER2 and CK for BCa's and HC's are shown.

In order to explore the capability of CEER to interrogate CTCs found in metastatic breast cancer patients, whole blood samples (N = 113) were analyzed from 60 healthy controls (HCs) and 53 metastatic BCa patients (from both 07ONC2 and 08ONC02 cohorts). Reference values were determined based on data obtained from HC subjects. The HER2 expression or activation distributions in all samples are shown in Figure 5. The reference values were set from HC mean plus 2.3 × SD. The differences between clinical samples and HCs were statistically significant (p < 0.01). The p-values were calculated using a two-sided Wilcoxon rank sum test. Based on this analysis, we determined that the overall average levels of phosphorylated HER2 in CTCs derived from metastatic breast cancer patients was 4.25-fold higher than those present in the healthy controls.

To determine pHER2 and total HER2 status in each sample, the background was subtracted and the control normalized signals were compared to the reference values for each marker. The CU values for pHER2 and total HER2 greater than the reference values are presented in Table 2. Out of 27 Stage 3/4 BCa patients (07ONC2 cohort), 17 CTC-samples were collected from metastatic BCa patients with a primary HER2-IHC negative status. 41% (7/17) patients with metastatic BCa and with a primary HER2-negative status displayed detectable HER2 expression in their CTCs which is consistent with published reports [31-39]. All of these CTCs from initial HER2-IHC negative patients also showed evidence of HER2 activation (pHER2) as detected by CEER. Interestingly, we also detected HER2 activation even without apparent HER2 over-expression in 18% (3/17) of CTCs from primary HER2-negative BCa patients. Within the group of relapsed BCa patients with primary HER2-positive tumors, 60% of the collected CTC samples (6/10) still showed HER2 expression. However, only 4 of the HER2-positive CTC samples displayed an activated HER2 status.

**Table 2:** Observation of HER2 conversion in circulating tumor cells

Sample	Primary IHC - HER2	pHER2	tHER2
A01-002	Positive	**1.9**	**5.8**
		
A01-003		**1.7**	**5.8**
		
A01-006		1.6	**5.8**
		
A01-014		**1.7**	**7.3**
		
A01-041		1.4	**7.2**
		
A02-027		0.1	ND
		
A02-034		**3.9**	**5.9**
		
A02-035		1.2	4.1
		
A02-036		0.1	1.7
		
A03-019		0.9	2.9

A02-039	Negative	1.1	ND
		
A02-052		**2.1**	**7.0**
		
A02-053		0.1	3.2
		
A02-056		0.5	ND
		
A03-008		0.7	ND
		
A01-019		**1.8**	**5.9**
		
A01-024		**1.8**	3.2
		
A01-028		**3.1**	4.0
		
A01-030		ND	ND
		
A01-034		**1.7**	**5.9**
		
A01-042		0.3	2.5
		
A02-001		0.7	ND
		
A02-017		**2.2**	1.1
		
A02-021		**2.1**	**6.9**
		
A02-028		**6.3**	**19.4**
		
A02-029		**3.9**	**6.0**
		
A02-031		**4.6**	**6.6**

A total of 27 breast cancer samples analyzed for their HER2 expression and activation are shown in the table. 17 blood samples were obtained from metastatic breast cancer patients with primary HER2-IHC negative status. Up to 59% (10/17) of initial HER2-IHC negative samples showed evidence of HER2 activation (pHER2) in their CTCs (**Bold**). CTCs with HER2 expression (tHER2) were found in 7 (**Bold**) out of 17 (41%) blood samples obtained from metastatic breast cancer patients with primary HER2 negative status. Levels of HER2 expression and phosphorylation are shown in CU.

An additional interesting observation was revealed from our CEER-based analysis of total and activated HER2 in the CTCs of metastatic breast cancer patients. While the levels of pHER2 in the CTCs collected from primary IHC-HER2-positive (HER2 status determined by IHC or FISH) patients who were still on trastuzumab therapy were significant; overall they were lower in comparison to the pHER2 levels observed in the CTCs derived from the primary IHC-HER2-negative patients. As described earlier, CTCs obtained from primary IHC-HER2-negative patients showed evidence of gaining a HER2-positive status. Taken together, our data demonstrate that discordance in HER2 status between the primary tumor and CTCs is not only at the level of HER2 expression but is also in terms of HER2 activation. One patient in particular, (A02-028), with an extremely high HER2 expression (19.4 CU) showed pHER2 level of 6.3CU. The pHER2/HER2 signal ratio in this patient is 0.32 (6.3/19.4), which is slightly higher than the typical pHER2/HER2 ratio of BT474 type cells (0.25). Because of the initial primary tumor HER2 status being negative, patients such as these will most likely not receive any HER2-targeted therapies.

In addition to detection of HER2 expression, we were also able to detect cytokeratins (pan-CK) in CTC samples. The level of CK correlates with the amount of CTCs in general, and the CKs in the CTCs of BCa patients were significantly higher than the healthy controls (Figure 5). However, CTCs stemming from different subclasses of breast cancer may have different levels of CK (100 MDA-MB-468 cells showed less than 9.7 CU whereas 10 SKBr3 cells showed 6.1 CU - data not shown). In addition, the type of CK expressed also varies in each tumor with different tissues of origin [40]. Therefore, CK values may not serve as an absolute quantitative reference for the isolated CTCs. Furthermore, recent studies demonstrating the contribution of EMT-derived mesenchymal cells to the CTC populations [41] may further hamper the utility of CKs in enumerating CTCs. Therefore, caution needs to be exercised in relying solely on CKs as a reference marker for enumerating CTCs; however, this issue will most likely be resolved in the future with better CTC isolation strategies. Nonetheless, CEER can be easily adapted to interrogate markers that can be used to enumerate the CTCs in addition to the active signaling pathways within them.

## Discussion

Development of companion diagnostics for targeted therapeutics in oncology is an urgent need to streamline efficient patient selection. Although interrogation of the primary tumor in order to determine potential responsiveness to a targeted therapy is becoming frequent; changes in the pattern of target expression, activation and downstream signaling as the tumor progresses from initial diagnosis to recurrence of metastatic disease are virtually never assessed. This is important as tumors are heterogenous and dynamic which results in their molecular circuitry being constantly modified with progression and treatment. To achieve this overarching goal, there has been aggressive progress in several non-invasive or minimally invasive tissue sampling methodologies in the past few years; however, meaningful molecular characterization of these limited amounts of clinical materials is challenging and is lagging behind.

We describe the development and application of a highly sensitive, novel proteomic technology, CEER, which can perform phosphorylation-driven analysis of many proteins in small amounts of clinical samples in a multiplexed manner. As demonstrated in this manuscript, CEER can be performed on rare CTCs isolated from blood or on limited number of tumor cells procured through FNAs. The multiplexed CEER technology can facilitate longitudinal monitoring of tumor progression or therapeutic response outcomes. The ability to quantitate the target protein activation state permits an additional evaluation of the signal transduction proteins beyond mere expression, potentially further predicting the utility of various targeted therapies.

CEER utilizes the formation of a unique immuno-complex requiring the co-localization of two detector enzyme-conjugated-antibodies once specific antibodies on the microarray surface have captured the target proteins. The channeling events between two detector enzymes with high turnover numbers (10^5^/min for GO and 10^4^/min for HRP [42,43]) in proximity enable the profiling of the RTK expression and activation in a highly sensitive manner. Besides enhancing the analytical specificity, the unique triplex assay configuration of CEER overcomes the issues associated with cross-reactivity found in a typical multiplexed immunoassay. This is particularly useful in the detection of protein phosphorylations as phosphorylation-specific antibodies are notorious for non-specifically binding to closely related analytes [44-46]. Furthermore, the methodology is flexible and assay components specific for additional targets can be easily added into the multiplexed format with minimal disruption. Indeed, there are other technologies in development for targeted proteomic analysis of clinical samples such as Reverse Phase Protein Arrays (RPPA) [47-49], Phospho-Flow cytometry [50] and ELISA [51]. They each have their specific advantages and shortcomings that are summarized in Table 3. However, CEER is the first described technique that incorporates the advantages of all of these techniques while providing ultra high sensitivity and specificity at a single cell level. Perhaps the only limitation of CEER is the need for screening compatible yet distinct triplex antibody sets for each target. However, with well-developed antibody production technologies, generating multiple antibodies specific for a target protein is not an obstacle for CEER development. As demonstrated in this manuscript, we have been successful in the simultaneous detection of protein expression and activation of EGFR, HER2, HER3, IGF1-R, c-MET, c-KIT, PI3K, Shc and other downstream signal transduction proteins with extreme sensitivity and specificity.

**Table 3:** Characteristics of CEER vs. Other Commonly Used Immunoassays

	CEER	Reverse Phase Protein Array	PhosphoFlow	ELISA
**Sample Protein Requirement**	Native proteins	Denatured proteins	Fixed proteins	Native or Fixed proteins

**Minimum Cell Requirement (Assay Sensitivity)**	1 cell	2500~15000 cells	1 × 10^4^~1 × 10^6 ^cells	150 to 300 cells

**Multiplex Format**	One sample, multiple analytes	Multiple samples, one analyte	One sample, multiple analytes (limited)	Multiple samples, one analyte

**Assay Specificity**	• High	• Low	• Low	• Medium

**Advantages of Assay**	• Simultaneous analysis of multiple analytes (e.g. selected analytes of a signaling pathway) on a single sample. • Suitable for clinical samples of rare cells as well as limited solid tissue samples.	• Simultaneous analysis of multiple samples for a single analyte. • Suitable for samples of cells or solid tissue.	• Detection of target molecules within a cell population in one sample. • Suitable for blood sample analysis.	• High throughput.

**Disadvantages of Assay**	• Screening compatible antibody sets for each target.	• Need to print each sample; not suitable for applications in clinical samples from individual patients. • Cross reaction of detector antibody.	• Not suitable for rare cell analysis or solid tissue samples. • Requires a large number of cells. • Requires samples in single cell suspension, limiting its use.	• Low sensitivity requires large numbers of cells. • Not suitable for rare cell analysis.

Using the CEER technology, we focused on analyzing the ErbB-RTK pathway activation profiles in both *HER2 *gene amplified and non-amplified breast cancer systems. HER2 signaling pathway forms a crucial driver of tumor growth in ~20-30% breast cancers. Breast cancer patients, whether HER2-positive or HER2-negative, develop recurrent metastatic disease with latency periods that can range from several years to even decades [52]. CTCs play a crucial role in tumor dissemination, and their monitoring during tumor dormancy periods can allow physicians to follow cancer changes over time as "real-time biopsies" [53,54]. CEER was used to successfully analyze CTCs isolated from metastatic breast cancer patients where the original primary tumors were identified as either HER2-positive or HER2-negative based on IHC analysis. Regardless of CTC isolation methods used, enriched CTC samples typically contain at least 10^4 ^or higher contaminating blood cells. As CEER generates a signal only when binding partners for specific corresponding epitopes are in proximity, it provides a realistic clinical means to investigate rare cells present in a high non-target background cell population.

HER2 analysis in CTCs can have important clinical significance for HER-targeted therapies. HER2-positive CTCs are common in women with HER2-IHC-positive primary breast tumors [36] and they were detected as such using the CEER technology. Such patients may benefit from secondary HER2-targeted therapy in an adjuvant setting. More importantly, CEER established the presence of HER2-positive CTCs in metastatic breast cancer patients whose primary tumors were originally deemed as HER2-negative based on IHC or FISH analysis. This "HER2 conversion" event is consistent with a growing body of evidence that HER2-positive status can be acquired during disease recurrence or progression in breast cancer patients [31-39]. However, CEER provided an additional and important readout in that the HER2 expressed on CTCs was phosphorylated indicating that the HER2 pathway was active in these CTCs from metastatic breast cancer patients. Furthermore, the pHER2 expression levels in HER2-IHC-negative patients were higher than those detected in HER2-IHC-positive patients some of who were receiving trastuzumab therapy. It remains to be seen if the HER2 expression in HER2-positive CTCs would be responsive to trastuzumab treatment. The ability to detect the HER2 activation status in CTCs has important clinical implications in terms of monitoring therapeutic responses and for understanding the HER2 biology in HER2-IHC-negative, metastatic breast cancer patients. At this time we cannot rule out the possibility that some of the HER2-IHC-negative primary breast cancers may actually be false negatives due to the inherent inaccuracy in IHC and FISH techniques and that a more sensitive technology such as CEER would reveal these cancers to have significant levels of HER2 expression. This will be addressed in future studies when we have access to the primary tumors and CTCs after metastasis from the same patient.

CEER revealed an active HER2 pathway in CTCs from HER2-IHC-negative breast cancer patients. Besides the translational implications of this finding, it may provide insight into the mechanism of breast cancer metastasis. The underlying mechanism responsible for HER2 conversion in these patients is unclear. It may be the result of therapeutic or other selection pressures on the heterogeneous tumor cell population that lead to a shift in the ErbB kinase signaling circuitry in the CTCs. Alternatively, it could be due to selection of dormant cancer cells that are HER2-positive. Although unproven, it may also be provocative to suggest that conversion to an active HER2 state in CTCs represents a transition to or selection of a stem cell-like population. Recent evidence demonstrates that overexpression of HER2 in breast cancer cells increases the cancer stem cell population [55]. Regardless of the mechanism behind HER2-conversion, the presence of HER2 expression/activation in CTCs requires further attention.

Detection of any CTCs before initiation of first-line therapy in patients with metastatic breast cancer is predictive of a poorer progression-free and overall survival [1,13]. Having a sensitive method such as CEER to monitor the functional profile-pathway shift in CTCs during the course of therapy may serve as a diagnostic marker for evaluating therapeutic outcomes and may help in subsequent therapy selection decisions. This would greatly enhance the value of CTC-based therapy monitoring rather than a mere enumeration tool as it is currently. As we can treat isolated CTCs using relevant ligands, this technology can provide the "activation potential" for CTCs in their route to a potential metastatic site. As the relationship of HER2 gene status between the primary breast cancer and synchronous distant metastasis has been reported to be concordant by several groups and quite different by others [45,56-58], it will be important to determine the correlation between RTK status in CTCs versus primary and metastatic lesions. These studies are currently ongoing in our laboratories and will be reported in the future.

## Conclusions

As having the capacity to predict potential therapy responses based on functional pathway-profiling is crucial in treating cancer patients, CEER provides an ideal platform to analyze a comprehensive array of cancer-causing biomarkers in various biological specimens and samples even with a limited amount of materials available (such as CTCs). Due to the superior sensitivity and specificity of this unique assay design, it may now be possible to not only select patients before initiating therapy, but also to analyze serially collected samples to keep up with the "evolving" disease. This would allow for the determination of optimal therapy combinations or sequencing to maximize therapy outcome.

## Methods

### Multiplexed microarray printing

Capture Abs were printed on nitrocellulose-coated glass slides (FAST,^®^, Whatman) using contact printers (QArray, Genetix). The spot diameter was approximately 175 μm and printed slides were kept in a desiccated chamber at 4°C. Each spot included a tracking dye and specific capture Abs (mouse monoclonal against extra cellular domains, ECDs). Approximately 500 pL of capture Abs were printed in triplicate and at serial dilution concentrations of 1 mg/mL, 0.5 mg/mL, and 0.25 mg/mL (Figure 1c). Purified mouse-IgGs were printed as a negative control. Analytical calibration reactions were performed on 8 pads and internal quality control reactions on 2 pads. Each slide allows processing of up to 4 unknown patient samples.

### Antibody conjugation and purification

Target specific-Abs (mouse monoclonal against intracellular domains, ICDs or p-Tyr) and corresponding detector enzymes were activated with a bi-functional cross-linker, succinimidyl-4-(N-maleimidomethyl) cyclohexane-1-carboxylate (SMCC) to make detector Ab-conjugates. The conjugates were purified by HPLC using a size-exclusion column. The Ab activities in the purified conjugates were detected by competition ELISA and enzyme activity was detected by a functional assay specific for each detector enzyme.

### Collaborative Enzyme Enhanced Reactive-immunoassay (CEER)

Slides were rinsed 2 times with TBST (50 mM Tris/150 mM NaCl/0.1% Tween-20, pH 7.2-7.4), blocked with 80 μL of Whatman Blocking Buffer for 1 hr at room temperature (RT), and then washed 2 times with TBST. Serially diluted cell lysate controls in 80 μL of dilution buffer (2% BSA/0.1% Triton X-100/TBS, pH 7.2-7.4) and samples were added to sub-arrays designated for standards on the slide and incubated for 1 hour (hr) at RT (Figure 1c). After incubation, slides were washed 4 times, 3 minutes (min) each time. The detector Abs were added in 80 μL of the reaction buffer and incubated for 2 hrs at RT. Unbound secondary detector Abs were removed by washing with TBST. The activation state-independent Abs were conjugated with the channeling enzyme, GO, and with the signal amplification moiety, HRP. When GO is supplied with a substrate such as glucose, it generates hydrogen peroxide (H_2_O_2_). When HRP is in close proximity to GO, H_2_O_2 _is channeled to HRP where it forms a stable complex. The HRP-H_2_O_2 _complex generates an amplified signal using a fluorogenic substrate such as tyramide to generate a reactive tyramide radical that covalently binds nearby nucleophilic residues. 80 μL of biotin-tyramide (400 μg/mL in ethanol, Perkin Elmer Life Science) at 5 μg/ml in 50 mM glucose/PBS was added to each pad and incubated for 15 min in the dark, then washed with TBST 4 times for 3 min each. The local deposition of activated biotin-tyramide was detected upon the addition of streptavidin (SA)-labeled Alexa647 (in PBS, Invitrogen) at 0.5 μg/ml (1:4000 dilution) in 2% BSA/0.1% Triton/TBS for 40 min. Upon completion of incubation, slides were washed 4 times with TBST, dried and kept in the dark until scanning on the microarray scanner.

### Cell line samples

SKBr3, MDA-MB-468, T47D and BT474 cell lines with varying degrees of ErbB-RTK expression [17-19] were obtained from ATCC and grown at 37°C in 5% CO_2 _for SKBr3 (McCoy's 5A medium with 10% FBS), MDA-MB-468 (Dulbecco's minimal essential medium, DMEM, 10% FBS), BT474 (DMEM, 10% FBS), and T47D (RPMI 1640, 10% FBS, 0.2 U/ml bovine insulin). Cells were harvested, counted and washed with 1× PBS, then stimulated with 100 nM epidermal growth factor (EGF) or transforming growth factor a (TGFα), 20 nM heregulin β (HRGβ) or both in serum-free growth media for 5 min. When Insulin-like Growth factor-1 (IGF1) was used, cells were treated with 100 ng/mL of IGF1 for 15 min. Stimulated cells were washed with 1× PBS and then lysed (lysis buffer: 50 mM Tris, pH 7.4, 150 mM NaCl, 1% Trition X-100 and 2 mM Na_3_VO_4_) and kept on ice for 30 min before taking the supernatant for a subsequent assay or kept at -80°C.

### Clinical blood samples

Blood samples from cancer patients (N = 53) and healthy controls (HCs) (N = 60) were collected according to the IRB approved protocol and informed consent obtained. Prior history of cancer or other serious chronic diseases were excluded from HCs. Specimens were shipped within 24 hrs and processed the same day, with resulting lysates stored at -80°C. All samples were taken from adult subjects (> 18 to < 88 years [yrs]) and sourced from multiple CRO sites. Diagnosis was performed according to RECIST (Response Evaluation Criteria in Solid Tumors). Whole blood from patients with histologically confirmed solid carcinoma with regional lymph node or distant metastases (Stage 3b or 4 - cohort 07ONC02, N = 27) were collected. Subjects with Stage 3b breast carcinoma had region lymph node staging of N1, N2, or N3. Samples were collected regardless of their therapy status. Whole blood samples from patients with progressive, evaluable metastatic stage IV breast cancer, and who were about to start systemic therapy (cohort 08ONC02, N = 26) were collected at base line. Both cohorts had similar age distributions with a baseline mean age of 57 ± 13 yrs. Extent of disease in both cohorts was determined by physical examination and imaging studies as per the primary physician. The tests utilized included one or more of the following: bone scans, PET/CT scans, CT of the abdomen, chest radiograph and/or CT of the chest for visceral metastases, sonogram and/or MRI for soft tissue disease. For CTC evaluation, 7.5 ml of blood samples were drawn into 10-ml evacuated ethylenediamine tetraacetic acid (EDTA) tubes. The CellSearch System (Veridex) was used for immuno-magnetic CTC isolation according to the protocol previously described using ferrofluids conjugated to Ab against epithelial cell adhesion molecule [59]. Enriched CTCs from blood were stimulated as described above.

### Tissue sample collection

Flash frozen breast cancer tissues were obtained from patients with ductal carcinoma at stage II or III (ILS Bio). HER2-IHC status was available for all samples. Xenograft models were generated using human breast cancer cell lines by subcutaneous injection into nude mice. When tumor size reached 400 mm^3^, tissue samples were collected by passing a 23 gauge needle attached to an evacuated syringe through each tumor 5 times. Collected samples from frozen tissues and xenografts were lysed in 100 μl of lysis buffer. Lysed samples were kept on ice for 30 min and centrifuged. Protein concentrations of supernatants were determined by BCA protein assay kit (Pierce), and the resulting lysates were stored at -80°C before subsequent analysis.

### Western blotting

Cell lysates for each cell line were aliquoted into single use vials. The protein concentration was determined by BCA protein assay kit (Pierce). Samples were prepared with sample buffer containing β-mercaptoethanol, boiled for 5 min, cooled to RT and loaded onto a NuPage (Invitrogen) 4 - 12% gel. Upon completion, the separated proteins were transferred to a nitrocellulose membrane, then washed, blocked with 5% milk blotto, and incubated with the 1° then 2° Abs before the detection process using 5-Bromo-4-Chloro-3'-Indolyphosphate p-Toluidine Salt (BCIP) and Nitro-Blue Tetrazolium Chloride (NBT). For the immunoprecipitation-western (IP-W) process for HER2 in tissues, sample lysates were incubated with magnetic beads conjugated with antibodies against ICD of HER2 overnight on a rocker at 4°C. The immuno-magnetically enriched lysates were then processed as described above.

### Data analysis

Each slide was scanned at three photomultiplier (PMT) gain settings to increase the effective dynamic range. Background corrected signal intensities were averaged for replicate spots printed in triplicate. The relative fluorescence value of the respective reagent blank was subtracted from each sample. Several quality criteria were used to filter data from further analysis including limits on the spot footprint, coefficient of variation for spot replicates, overall pad background and the intensity of the reagent blank.

For each assay, a sigmoidal standard curve was generated from seven concentrations of serially diluted cell lysates prepared from cell lines MDA-MB-468 (HER1-positive) and SKBr3 (HER2-positive). Each curve was plotted as a function of signal intensity vs. log concentration derived units, CU (Computed Unit). The data were fit to a five parameter equation by nonlinear regression [60], simultaneously fitting all three dilutions of the capture Ab. Fitting was carried out using R, an open source statistical software package [61]. The individual predictions from each of the standard curves (3 capture Ab dilutions and 3 PMT gain-set scanning) were combined into a single, final prediction. The final prediction was calculated by a weighted (determined by the slope) average of the individual predictions and then designated as CU.

## List of Abbreviations

Abs: antibodies; BCa: breast cancer; BSA: bovine serum albumin; CEER: COllaborative Proximity ImmunoAssay; CT: computed tomography; CTCs: circulating tumor cells; CU: computed unit; DMEM: Dulbecco's minimal essential medium; EDTA: ethylenediamine tetraacetic acid; EGF: epidermal growth factor; ELISA: enzyme-linked immunosorbent assay; ErbB: erythroblastic leukemia viral oncogene homolog; FISH: Fluorescence In Situ Hybridization; FNA: fine needle aspirate; GO: glucose oxidase; HCs: healthy controls; HER1: human epidermal growth factor receptor 1; HER2: human epidermal growth factor receptor 2; HPLC: high performance liquid chromatography; HRG: Heregulin; HRP: Horse Radish Peroxidase; IGF: insulin-like growth factor; IGF1R:IGF1-receptor; IHC: immunohistochemistry; LOD: limit of detection; MET: mesenchymal-epithelial; mRNA: messenger RNA; OD: optical density; p95HER2: truncated HER2; PBS: phosphate buffered saline; PET: positron emission tomography; pHER: phosphorylated HER; PMT: photomultiplier; RFU: relative fluorescence unit; photomultiplier; RT: room temperature; RTKs: receptor tyrosine kinases; SMCC: succinimidyl-4-(N-maleimidomethyl) cyclohexane-1-carboxylate; TBST:Tris-Buffered Saline Tween-20; TGFα: transforming growth factor alpha; tHER: total expression of HER; TBS: tris buffered saline

## Competing interests

The authors declare that they have no competing interests.

## Authors' contributions

PK, XL, SS, BY directed research; PK, TL, XL, SS designed experiments; XL, TL, RB, RK, LL performed experiments; PK, XL, TL, RB, A., GL, SS, BY analyzed data; AK, GL developed algorithms; PK, BY, SS, SN wrote the manuscript. SS is Chief Investigator who conceived the study design. All authors read and approved the final manuscript.

## References

[B1] Slamon DJ Clark GM Wong SG Levin WJ Ullrich A McGuire WL Human breast cancer: correlation of relapse and survival with amplification of the HER-2/neu oncogene Science 1987 235 4785 177 182 10.1126/science.3798106 3798106

[B2] Slamon DJ Godolphin W Jones LA Holt JA Wong SG Keith DE Levin WJ Stuart SG Udove J Ullrich A Studies of the HER-2/neu proto-oncogene in human breast and ovarian cancer Science 1989 244 4905 707 712 10.1126/science.2470152 2470152

[B3] Dawood S Broglio K Buzdar AU Hortobagyi GN Giordano SH Prognosis of women with metastatic breast cancer by HER2 status and trastuzumab treatment: an institutional-based review J Clin Oncol 2010 28 1 92 98 10.1200/JCO.2008.19.9844 PMC2799236 19933921

[B4] Ferretti G Fabi A Felici A Papaldo P Improved prognosis by trastuzumab of women with HER2-positive breast cancer compared with those with HER2-negative disease J Clin Oncol 2010 28 20 e337 author reply e338-339 10.1200/JCO.2010.28.2525 20479395

[B5] Bast RC Jr Ravdin P Hayes DF Bates S Fritsche H Jr Jessup JM Kemeny N Locker GY Mennel RG Somerfield MR 2000 update of recommendations for the use of tumor markers in breast and colorectal cancer: clinical practice guidelines of the American Society of Clinical Oncology J Clin Oncol 2001 19 6 1865 1878 10.1200/JCO.2001.19.6.1865 11251019

[B6] Nahta R Esteva FJ HER2 therapy: molecular mechanisms of trastuzumab resistance Breast Cancer Res 2006 8 6 215 10.1186/bcr1612 PMC1797036 17096862

[B7] Paik S Shak S Tang G Kim C Baker J Cronin M Baehner FL Walker MG Watson D Park T A multigene assay to predict recurrence of tamoxifen-treated, node-negative breast cancer N Engl J Med 2004 351 27 2817 2826 10.1056/NEJMoa041588 15591335

[B8] Paik S Tang G Shak S Kim C Baker J Kim W Cronin M Baehner FL Watson D Bryant J Gene expression and benefit of chemotherapy in women with node-negative, estrogen receptor-positive breast cancer J Clin Oncol 2006 24 23 3726 3734 10.1200/JCO.2005.04.7985 16720680

[B9] Gown AM Current issues in ER and HER2 testing by IHC in breast cancer Mod Pathol 2008 21 Suppl 2 S8 S15 10.1038/modpathol.2008.34 18437174

[B10] Rhodes A Jasani B Barnes DM Bobrow LG Miller KD Reliability of immunohistochemical demonstration of oestrogen receptors in routine practice: interlaboratory variance in the sensitivity of detection and evaluation of scoring systems J Clin Pathol 2000 53 2 125 130 10.1136/jcp.53.2.125 PMC1763294 10767828

[B11] Reddy JC Reimann JD Anderson SM Klein PM Concordance between central and local laboratory HER2 testing from a community-based clinical study Clin Breast Cancer 2006 7 2 153 157 10.3816/CBC.2006.n.025 16800975

[B12] Cuadros M Villegas R Systematic review of HER2 breast cancer testing Appl Immunohistochem Mol Morphol 2009 17 1 1 7 10.1097/PAI.0b013e318169fc1c 18685491

[B13] Cristofanilli M Budd GT Ellis MJ Stopeck A Matera J Miller MC Reuben JM Doyle GV Allard WJ Terstappen LW Circulating tumor cells, disease progression, and survival in metastatic breast cancer N Engl J Med 2004 351 8 781 791 10.1056/NEJMoa040766 15317891

[B14] Hayes DF Cristofanilli M Budd GT Ellis MJ Stopeck A Miller MC Matera J Allard WJ Doyle GV Terstappen LW Circulating tumor cells at each follow-up time point during therapy of metastatic breast cancer patients predict progression-free and overall survival Clin Cancer Res 2006 12 14 Pt 1 4218 4224 10.1158/1078-0432.CCR-05-2821 16857794

[B15] Nagrath S Sequist LV Maheswaran S Bell DW Irimia D Ulkus L Smith MR Kwak EL Digumarthy S Muzikansky A Isolation of rare circulating tumour cells in cancer patients by microchip technology Nature 2007 450 7173 1235 1239 10.1038/nature06385 PMC3090667 18097410

[B16] Pachmann K Camara O Kavallaris A Krauspe S Malarski N Gajda M Kroll T Jorke C Hammer U Altendorf-Hofmann A Monitoring the response of circulating epithelial tumor cells to adjuvant chemotherapy in breast cancer allows detection of patients at risk of early relapse J Clin Oncol 2008 26 8 1208 1215 10.1200/JCO.2007.13.6523 18323545

[B17] Dragowska WH Warburton C Yapp DT Minchinton AI Hu Y Waterhouse DN Gelmon K Skov K Woo J Masin D HER-2/neu overexpression increases the viable hypoxic cell population within solid tumors without causing changes in tumor vascularization Mol Cancer Res 2004 2 11 606 619 15561777

[B18] Filmus J Trent JM Pollak MN Buick RN Epidermal growth factor receptor gene-amplified MDA-468 breast cancer cell line and its nonamplified variants Mol Cell Biol 1987 7 1 251 257 10.1128/mcb.7.1.251-257.1987 PMC365064 3494191

[B19] Imai Y Leung CK Friesen HG Shiu RP Epidermal growth factor receptors and effect of epidermal growth factor on growth of human breast cancer cells in long-term tissue culture Cancer Res 1982 42 11 4394 4398 6290036

[B20] Uherek C Tonn T Uherek B Becker S Schnierle B Klingemann HG Wels W Retargeting of natural killer-cell cytolytic activity to ErbB2-expressing cancer cells results in efficient and selective tumor cell destruction Blood 2002 100 4 1265 1273 12149207

[B21] Yang S Raymond-Stintz MA Ying W Zhang J Lidke DS Steinberg SL Williams L Oliver JM Wilson BS Mapping ErbB receptors on breast cancer cell membranes during signal transduction J Cell Sci 2007 120 Pt 16 2763 2773 10.1242/jcs.007658 17652160

[B22] Moasser MM Basso A Averbuch SD Rosen N The tyrosine kinase inhibitor ZD1839 ("Iressa") inhibits HER2-driven signaling and suppresses the growth of HER2-overexpressing tumor cells Cancer Res 2001 61 19 7184 7188 11585753

[B23] Nahta R Yuan LX Zhang B Kobayashi R Esteva FJ Insulin-like growth factor-I receptor/human epidermal growth factor receptor 2 heterodimerization contributes to trastuzumab resistance of breast cancer cells Cancer Res 2005 65 23 11118 11128 10.1158/0008-5472.CAN-04-3841 16322262

[B24] Hirai M Gamou S Minoshima S Shimizu N Two independent mechanisms for escaping epidermal growth factor-mediated growth inhibition in epidermal growth factor receptor-hyperproducing human tumor cells J Cell Biol 1988 107 2 791 799 10.1083/jcb.107.2.791 PMC2115197 2458359

[B25] Ross DT Scherf U Eisen MB Perou CM Rees C Spellman P Iyer V Jeffrey SS Van de Rijn M Waltham M Systematic variation in gene expression patterns in human cancer cell lines Nat Genet 2000 24 3 227 235 10.1038/73432 10700174

[B26] Wolff AC Hammond ME Schwartz JN Hagerty KL Allred DC Cote RJ Dowsett M Fitzgibbons PL Hanna WM Langer A American Society of Clinical Oncology/College of American Pathologists guideline recommendations for human epidermal growth factor receptor 2 testing in breast cancer J Clin Oncol 2007 25 1 118 145 10.1200/JCO.2006.09.2775 17159189

[B27] Paik S Kim C Wolmark N HER2 status and benefit from adjuvant trastuzumab in breast cancer N Engl J Med 2008 358 13 1409 1411 10.1056/NEJMc0801440 18367751

[B28] Roche PC Suman VJ Jenkins RB Davidson NE Martino S Kaufman PA Addo FK Murphy B Ingle JN Perez EA Concordance between local and central laboratory HER2 testing in the breast intergroup trial N9831 J Natl Cancer Inst 2002 94 11 855 857 10.1093/jnci/94.11.855 12048274

[B29] Cristofanilli M Broglio KR Guarneri V Jackson S Fritsche HA Islam R Dawood S Reuben JM Kau SW Lara JM Circulating tumor cells in metastatic breast cancer: biologic staging beyond tumor burden Clin Breast Cancer 2007 7 6 471 479 17386124

[B30] Riethdorf S Fritsche H Muller V Rau T Schindlbeck C Rack B Janni W Coith C Beck K Janicke F Detection of circulating tumor cells in peripheral blood of patients with metastatic breast cancer: a validation study of the CellSearch system Clin Cancer Res 2007 13 3 920 928 10.1158/1078-0432.CCR-06-1695 17289886

[B31] Apostolaki S Perraki M Pallis A Bozionelou V Agelaki S Kanellou P Kotsakis A Politaki E Kalbakis K Kalykaki A Circulating HER2 mRNA-positive cells in the peripheral blood of patients with stage I and II breast cancer after the administration of adjuvant chemotherapy: evaluation of their clinical relevance Ann Oncol 2007 18 5 851 858 10.1093/annonc/mdl502 17301075

[B32] Bozionellou V Mavroudis D Perraki M Papadopoulos S Apostolaki S Stathopoulos E Stathopoulou A Lianidou E Georgoulias V Trastuzumab administration can effectively target chemotherapy-resistant cytokeratin-19 messenger RNA-positive tumor cells in the peripheral blood and bone marrow of patients with breast cancer Clin Cancer Res 2004 10 24 8185 8194 10.1158/1078-0432.CCR-03-0094 15623593

[B33] Cao S Li Y Li J Li CF Zhang W Yang ZQ Meng SD Quantitative determination of HER2 expression by confocal microscopy assay in CTCs of breast cancer Oncol Rep 2010 23 2 423 428 20043103

[B34] Fehm T Muller V Aktas B Janni W Schneeweiss A Stickeler E Lattrich C Lohberg CR Solomayer E Rack B HER2 status of circulating tumor cells in patients with metastatic breast cancer: a prospective, multicenter trial Breast Cancer Res Treat 2010 124 2 403 412 10.1007/s10549-010-1163-x 20859679

[B35] Flores LM Kindelberger DW Ligon AH Capelletti M Fiorentino M Loda M Cibas ES Janne PA Krop IE Improving the yield of circulating tumour cells facilitates molecular characterisation and recognition of discordant HER2 amplification in breast cancer Br J Cancer 2010 102 10 1495 1502 10.1038/sj.bjc.6605676 PMC2869174 20461092

[B36] Ignatiadis M Rothe F Chaboteaux C Durbecq V Rouas G Criscitiello C Metallo J Kheddoumi N Singhal SK Michiels S HER2-positive circulating tumor cells in breast cancer PLoS One 2011 6 1 e15624 10.1371/journal.pone.0015624 PMC3018524 21264346

[B37] Meng S Tripathy D Shete S Ashfaq R Haley B Perkins S Beitsch P Khan A Euhus D Osborne C HER-2 gene amplification can be acquired as breast cancer progresses Proc Natl Acad Sci USA 2004 101 25 9393 9398 10.1073/pnas.0402993101 PMC438987 15194824

[B38] Riethdorf S Muller V Zhang L Rau T Loibl S Komor M Roller M Huober J Fehm T Schrader I Detection and HER2 expression of circulating tumor cells: prospective monitoring in breast cancer patients treated in the neoadjuvant GeparQuattro trial Clin Cancer Res 2010 16 9 2634 2645 10.1158/1078-0432.CCR-09-2042 20406831

[B39] Tewes M Aktas B Welt A Mueller S Hauch S Kimmig R Kasimir-Bauer S Molecular profiling and predictive value of circulating tumor cells in patients with metastatic breast cancer: an option for monitoring response to breast cancer related therapies Breast Cancer Res Treat 2009 115 3 581 590 10.1007/s10549-008-0143-x 18679793

[B40] Rakha EA Reis-Filho JS Ellis IO Basal-like breast cancer: a critical review J Clin Oncol 2008 26 15 2568 2581 10.1200/JCO.2007.13.1748 18487574

[B41] Armstrong AJ Marengo MS Oltean S Kemeny G Bitting RL Turnbull JD Herold CI Marcorn PK George DJ Garcia-Blanco MA Circulating Tumor Cells from Patients with Advanced Prostate and Breast Cancer Display Both Epithelial and Mesenchymal Markers Mol Cancer Res 2011 9 997 1007 10.1158/1541-7786.MCR-10-0490 PMC3157566 21665936

[B42] Gibson QH Swoboda BE Massey V Kinetics and Mechanism of Action of Glucose Oxidase J Biol Chem 1964 239 3927 3934 14257628

[B43] Klapper MH Hackett DP The Oxidatic Activity of Horseradish Peroxidase. I. Oxidation of Hydro- and Naphthohydroquinones J Biol Chem 1963 238 3736 3742 14109213

[B44] Greenberg JI Shields DJ Barillas SG Acevedo LM Murphy E Huang J Scheppke L Stockmann C Johnson RS Angle N A role for VEGF as a negative regulator of pericyte function and vessel maturation Nature 2008 456 7223 809 813 10.1038/nature07424 PMC2605188 18997771

[B45] Grupka NL Lear-Kaul KC Kleinschmidt-DeMasters BK Singh M Epidermal growth factor receptor status in breast cancer metastases to the central nervous system. Comparison with HER-2/neu status Arch Pathol Lab Med 2004 128 9 974 979 10.5858/2004-128-974-EGFRSI 15335267

[B46] Mellberg S Dimberg A Bahram F Hayashi M Rennel E Ameur A Westholm JO Larsson E Lindahl P Cross MJ Transcriptional profiling reveals a critical role for tyrosine phosphatase VE-PTP in regulation of VEGFR2 activity and endothelial cell morphogenesis FASEB J 2009 23 5 1490 1502 10.1096/fj.08-123810 19136612

[B47] Aguilar-Mahecha A Cantin C O'Connor-McCourt M Nantel A Basik M Development of reverse phase protein microarrays for the validation of clusterin, a mid-abundant blood biomarker Proteome Sci 2009 7 15 10.1186/1477-5956-7-15 PMC2672067 19348683

[B48] Paweletz CP Charboneau L Bichsel VE Simone NL Chen T Gillespie JW Emmert-Buck MR Roth MJ Petricoin IE Liotta LA Reverse phase protein microarrays which capture disease progression show activation of pro-survival pathways at the cancer invasion front Oncogene 2001 20 16 1981 1989 10.1038/sj.onc.1204265 11360182

[B49] Rapkiewicz A Espina V Zujewski JA Lebowitz PF Filie A Wulfkuhle J Camphausen K Petricoin EF Liotta LA Abati A The needle in the haystack: application of breast fine-needle aspirate samples to quantitative protein microarray technology Cancer 2007 111 3 173 184 10.1002/cncr.22686 17487852

[B50] Krutzik PO Irish JM Nolan GP Perez OD Analysis of protein phosphorylation and cellular signaling events by flow cytometry: techniques and clinical applications Clin Immunol 2004 110 3 206 221 10.1016/j.clim.2003.11.009 15047199

[B51] Matsumoto T Schiller P Dieterich LC Bahram F Iribe Y Hellman U Wikner C Chan G Claesson-Welsh L Dimberg A Ninein is expressed in the cytoplasm of angiogenic tip-cells and regulates tubular morphogenesis of endothelial cells Arterioscler Thromb Vasc Biol 2008 28 12 2123 2130 10.1161/ATVBAHA.108.169128 18772498

[B52] Meng S Tripathy D Frenkel EP Shete S Naftalis EZ Huth JF Beitsch PD Leitch M Hoover S Euhus D Circulating tumor cells in patients with breast cancer dormancy Clin Cancer Res 2004 10 24 8152 8162 10.1158/1078-0432.CCR-04-1110 15623589

[B53] de Bono JS Scher HI Montgomery RB Parker C Miller MC Tissing H Doyle GV Terstappen LW Pienta KJ Raghavan D Circulating tumor cells predict survival benefit from treatment in metastatic castration-resistant prostate cancer Clin Cancer Res 2008 14 19 6302 6309 10.1158/1078-0432.CCR-08-0872 18829513

[B54] Scher HI Jia X de Bono JS Fleisher M Pienta KJ Raghavan D Heller G Circulating tumour cells as prognostic markers in progressive, castration-resistant prostate cancer: a reanalysis of IMMC38 trial data Lancet Oncol 2009 10 3 233 239 10.1016/S1470-2045(08)70340-1 PMC2774131 19213602

[B55] Korkaya H Paulson A Iovino F Wicha MS HER2 regulates the mammary stem/progenitor cell population driving tumorigenesis and invasion Oncogene 2008 27 47 6120 6130 10.1038/onc.2008.207 PMC2602947 18591932

[B56] Tanner M Jarvinen P Isola J Amplification of HER-2/neu and topoisomerase IIalpha in primary and metastatic breast cancer Cancer Res 2001 61 14 5345 5348 11454672

[B57] Tapia C Savic S Wagner U Schonegg R Novotny H Grilli B Herzog M Barascud AD Zlobec I Cathomas G HER2 gene status in primary breast cancers and matched distant metastases Breast Cancer Res 2007 9 3 R31 10.1186/bcr1676 PMC1929093 17511881

[B58] Zidan J Dashkovsky I Stayerman C Basher W Cozacov C Hadary A Comparison of HER-2 overexpression in primary breast cancer and metastatic sites and its effect on biological targeting therapy of metastatic disease Br J Cancer 2005 93 5 552 556 10.1038/sj.bjc.6602738 PMC2361603 16106267

[B59] Fehm T Sagalowsky A Clifford E Beitsch P Saboorian H Euhus D Meng S Morrison L Tucker T Lane N Cytogenetic evidence that circulating epithelial cells in patients with carcinoma are malignant Clin Cancer Res 2002 8 7 2073 2084 12114406

[B60] Ritz C Streibig J Bioassay analysis using R Journal of Statistical Software 2005 12 1 22

[B61] Development Core Team R A language and environment for statistical computing 2008 R Foundation for Statistical Computing, Vienna, Austria

